# Pharmacokinetic Characteristics, Tissue Bioaccumulation and Toxicity Profiles of Oral Arsenic Trioxide in Rats: Implications for the Treatment and Risk Assessment of Acute Promyelocytic Leukemia

**DOI:** 10.3389/fphar.2021.647687

**Published:** 2021-05-28

**Authors:** Wensheng Liu, Bin Wang, Yilei Zhao, Zhiqiang Wu, Andi Dong, Hongzhu Chen, Liwang Lin, Jing Lu, Xin Hai

**Affiliations:** Department of Pharmacy, The First Affiliated Hospital of Harbin Medical University, Harbin, China

**Keywords:** pharmacokinetics, tissue bioaccumulation, arsenic toxicity, acute promyelocytic leukemia, dimethylated arsenic, monomethylated arsenic, oral arsenic trioxide

## Abstract

Oral arsenic trioxide (ATO) has demonstrated a favorable clinical efficiency in the treatment of acute promyelocytic leukemia (APL). However, the pharmacokinetic characteristics, tissue bioaccumulation, and toxicity profiles of arsenic metabolites *in vivo* following oral administration of ATO have not yet been characterized. The present study uses high performance liquid chromatography-hydride generation-atomic fluorescence spectrometry (HPLC-HG-AFS) to assess the pharmacokinetics of arsenic metabolites in rat plasma after oral and intravenous administration of 1 mg kg^−1^ ATO. In addition, the bioaccumulation of arsenic metabolites in blood and selected tissues were evaluated after 28 days oral administration of ATO in rats at a dose of 0, 2, 8, and 20 mg kg^−1^ d^−1^. The HPLC-HG-AFS analysis was complemented by a biochemical, hematological, and histopathological evaluation conducted upon completion of ATO treatment. Pharmacokinetic results showed that arsenite (As^III^) reached a maximum plasma concentration rapidly after initial dosing, and the absolute bioavailability of As^III^ was 81.03%. Toxicological results showed that the levels of aspartate aminotransferase (AST), alanine aminotransferase (ALT), and white blood cells (WBC) in the 20 mg kg^−1^ d^−1^ ATO group were significantly increased compared to the control group (*p* < 0.05). The distribution trend of total arsenic in the rat was as follows: whole blood > kidney > liver > heart. Dimethylated arsenic (DMA) was the predominant bioaccumulative metabolite in the whole blood, liver, and heart, while monomethylated arsenic (MMA) was the predominant one in the kidney. Collectively, these results revealed that oral ATO was rapidly absorbed, well-tolerated, and showed organ-specific and dose-specific bioaccumulation of arsenic metabolites. The present study provides preliminary evidence for clinical applications and the long-term safety evaluation of oral ATO in the treatment of APL.

## Introduction

Arsenic trioxide (ATO) is a well-known toxic metalloid that has been used for treating various diseases such as ulcers, the plague, and malaria for more than two millennia (Emadi and Gore, 2010). In the 1970s, researchers from Harbin Medical University (China) found that intravenous (i.v.) ATO has a significant positive therapeutic effect on acute promyelocytic leukemia (APL) (Zhang et al., 1996). Currently, i.v. ATO-based regimens have become the backbone of treatment for newly diagnosed or relapsed APL and led to 86–100% of patients achieving complete remission (CR) (Mathews et al., 2010; Lo-Coco et al., 2013; Burnett et al., 2015). In general, ATO is administrated by i.v. infusion over 2–3 h in a dose of 10 mg or 0.15 mg kg^−1^ daily until CR (Mathews et al., 2010; Lo-Coco et al., 2013; Burnett et al., 2015). However, long-term i.v. ATO is resource-demanding, inconvenient and costly, which makes i.v. ATO treatment challenging (Kumana et al., 2020). Oral administration of ATO appears to be an alternatively practicable option to the management of APL that has shown similar efficacy to i.v. ATO (Gill et al., 2018; Kumana et al., 2020; Ravandi et al., 2020; Sasijareonrat et al., 2020). Regardless of the route of administration, the risks of ATO treatment raises concerns, due to potential adverse reactions, e.g., QTc prolongation, liver injury, leukocytosis, and renal failure (Lo-Coco et al., 2013; Gill et al., 2018; Sasijareonrat et al., 2020).

Increasing evidence from clinical and laboratory studies demonstrates that how ATO exerts its pharmacological activities and initiates the toxicological process mainly depends on the generation of various active metabolic intermediates (Khaleghian et al., 2014; Khairul et al., 2017; Lu et al., 2019). Generally, when i.v. or oral administration, arsenite (As^III^, the hydrolyzed form of ATO) is subjected to sequential reduction and oxidative methylation reactions in the blood to form arsenate (As^V^), monomethylated arsenic (MMA), and dimethylated arsenic (DMA) (Cullen, 2014; Hirano, 2020). Previously, the methylation metabolism of arsenic was described as a detoxification process. However, more recent evidence has established that the intermediate metabolites of arsenic, trivalent forms of MMA and DMA (MMA^III^, DMA^III^), are more toxic than inorganic arsenic (iAs, including As^III^ and As^V^) (Khairul et al., 2017; Hirano, 2020). Trivalent arsenicals show high binding affinity to thiol groups of free cysteine residues, leading to structural modifications of proteins as well as enzyme inhibition (Shen et al., 2013; Soria et al., 2017). Comparatively, the pentavalent forms of MMA and DMA (MMA^V^, DMA^V^) are the predominant arsenic methylated metabolites in urine, which are considered a tumor promoter and significantly associated with adverse health effects (Wang et al., 2013; Kuo et al., 2017). Meanwhile, the plasma arsenic metabolites are mainly present in a free state and make them distributed to different organs. In addition, the combination of arsenic metabolites and proteins in erythrocytes will result in its accumulation in organs (Shen et al., 2013). Therefore, the tissue distribution and bioaccumulation of arsenic and its intermediate metabolites also have a great potential for determining its actions as “toxicant” (Pei et al., 2019; Yi et al., 2020). However, most existing studies report on the tissue distribution and bioaccumulation of total arsenic (TAs) concentration (Fazio et al., 2019; Wu et al., 2020) and studies focusing on the pharmacokinetic characteristics, tissue bioaccumulation, and toxicity profile of iAs and its methylation metabolites (MMA and DMA) in blood and organs following the oral administration of ATO are currently limited.

In the present study, high-performance liquid chromatography-hydride generation-atomic fluorescence spectrometry (HPLC-HG-AFS) was applied to determine the content of various arsenic metabolites (As^III^, As^V^, MMA, DMA) in plasma to elucidate the pharmacokinetic characteristics of oral ATO. Furthermore, the determination of arsenic metabolite content in tissues, histopathology, biochemistry, and hematology examination were also undertaken to elaborate on the potential biological and toxicological outcomes of arsenic metabolites following orally administrated ATO in different dose ranges for 28 days. Taken together, the results reported here could provide novel insights into developing new ATO formulations optimized for oral administration and provide a preliminary basis for subsequent evaluations of the sub-chronic safety and risk assessment of oral ATO in the treatment of APL.

## Materials and Methods

### Chemicals and Reagents

ATO powder (purity 99.9%) was obtained from Harbin Yida Pharmaceutical Co., Ltd. (Harbin, China). Standard compounds of arsenite (As^III^, GBW08666), arsenate (As^V^, GBW08667), monomethylarsonic acid (MMA^V^, GBW08668), and dimethylarsinic acid (DMA^V^, GBW08669) were purchased from the National Institute of Metrology (Beijing, China). The sodium acetate (CH_3_COONa), sodium dihydrogen phosphate (NaH_2_PO_4_), potassium nitrate (KNO_3_), disodium ethylenediaminetetraacetic acid (EDTA-2Na), and potassium hydroxide (KOH) were of guarantee grade and were all purchased from Sinopharm Chemical Reagent Co., Ltd. (Shanghai, China). Perchloric acid (HClO_4_, purity: 72%), ammonia (purity: 25%), and potassium borohydride (KBH_4_) were supplied by Kermel Chemical Reagent Co., Ltd. (Tianjin, China). Hydrogen peroxide (H_2_O_2_, purity:30%) was purchased from Jinxi Chemical Plant (Tianjin, China). Ethyl carbamate was purchased from Guangfu Fine Chemical Research Institute (Tianjin, China). Nitric acid (HNO_3_, purity: 70%), and hydrochloric acid (HCl, purity: 37%) were all purchased from Sigma (Sigma-Aldrich Chemical Co., United States). The deionized water was prepared using a Milli-Q water purification system (Thermo Genpure, United States). The ATO solution (1 mg ml^−1^) and stock solution of arsenic standard compounds (1 μg ml^−1^) were prepared with fresh deionized water and stored in the dark at 4°C. Saline solution was obtained from Sanlian Pharmaceutical Co., Ltd. (Harbin, China).

### Experimental Animals

The animal experimental procedures and protocols were conducted according to the Guide for the Care and Use of Laboratory Animals (NIH Publications No. 8023, revised 1978), and were approved by the Animal Ethics Committee of The First Affiliated Hospital of Harbin Medical University. Healthy male and female Sprague-Dawley (SD) rats (5–6 weeks old) were obtained from the Experimental Animal Center of Harbin Medical University (Harbin, China, Certificate No. SCXK (Hei) 2013-001). The animals were acclimated for at least 1 week and fasted overnight with free access to water before the experiments. At the beginning of the study (i.e., at an age of 6–7 weeks), body weights of about 200 g were determined. During the experimental period, fixed-formula rat granular food and water were provided *ad libitum*, and the environmental conditions were maintained at 22 ± 3°C and 50 ± 15% relative humidity, 12 h light/dark cycles, and 12 air change cycles/h.

### Pharmacokinetic Study by Single Administration

ATO powders were dissolved in 0.9% saline solution to obtain the 1 mg ml^−1^ stock solution. Ten male SD rats were randomly and equally divided into intragastric (i.g.) and i.v. administration ATO group (*n* = 5, respectively). Administered dosages for the experimental animals were estimated based on the body surface area dosage conversion factors multiplying the dosage used in humans (Nair et al., 2018). Thus, the selected ATO dose of 1 mg kg^−1^ is equivalent to a human therapeutic dose of ATO in APL treatment (0.15 mg kg^−1^ d^−1^) and was administered at a single dose to each rat with a microinjector or injector.

Blood samples of about 0.5 ml were collected from the tail vein into heparinized 1.5 ml polythene tubes at 5, 10, 15, 30, and 45 min, 1, 1.5, 2, 4, 6, 8, 12, and 24 h after ATO administration. Plasma was separated by centrifugation at 19,090 × *g* for 5 min (Centrifuge 5430 R, Eppendorf) and stored at −80°C until analysis.

### Toxicity Profiles and Tissue Bioaccumulation of Total Arsenic and Arsenic Metabolites Following Oral Arsenic Trioxide for 28 Days

#### Administration and Sampling

Forty-eight (24 male, 24 female) rats were randomly divided into four groups (*n* = 12 for each group), with the same average body weight and compromising half male and female: 1) control group, 2) dose of 2 mg kg^−1^ d^−1^ATO (2 times of the clinical dosage), 3) dose of 8 mg kg^−1^ d^−1^ ATO (8 times of the clinical dosage), and 4) dose of 20 mg kg^−1^ d^−1^ ATO (20 times of the clinical dosage). Saline solution (control group) or ATO (treated group) was administered intragastrically once a day for 28 days. Throughout the study, the changes in clinical presentation and mortality of rats were monitored daily. Additionally, individual body weight was recorded every 3 days during the ATO administration period.

One day after the last administration, the rats were fasted overnight and anesthetized by intraperitoneal injection with 20% urethane (5 ml kg^−1^). Blood samples were collected from the abdominal aorta into tubes for biochemistry and hematology analysis. Thereafter, tissue samples were rinsed with 0.9% saline solution to remove the blood, dried with filter paper, weighed, and stored at −80°C until subsequent analysis.

#### Biochemistry and Hematology Examination

Biochemistry and hematology examination were conducted at the Central Laboratory of The First Affiliated Hospital of Harbin Medical University, Harbin, China. Total protein (TP), alanine aminotransferase (ALT), aspartate aminotransferase (AST), glucose (GLU) measurements in the serum were analyzed using a Beckman Coulter AU5800 automatic biochemistry analyzer (Beckman Coulter, Inc., United States). The hematology examination was assessed using Mindray BC6900 automatic blood cell analyzer (Mindray, Shenzhen, China), including white blood cells (WBC), red blood cells (RBC), hemoglobin (HGB), neutrophils absolute value (NeU), and platelets (PLT).

#### Histopathological Examination

The rat tissue samples (liver, kidney, heart) were fixed with 4% paraformaldehyde for 48 h. Tissues were processed for paraffin wax embedding, sectioned into 4–6 μm slices, and stained with standard hematoxylin and eosin (H&E). Thereafter, slices of the tissues were examined with an optical microscope (magnification ×400) (Leica, Germany).

#### Total Arsenic and Arsenic Metabolites Determination

The extractable arsenic metabolites were separated and measured using an HPLC-HG-AFS (LC-AFS 6500, Beijing Haiguang Instrument Co., Ltd., Beijing, China) equipped with a Hamilton PRP-X100 HPLC column (250 mm × 4.1 mm, 10 μM particle size). The determination of TAs and arsenic metabolites in tissue and blood samples was performed according to the methods described by Guo et al. with minor modifications as described below (Guo et al., 2017).

For determining the bioaccumulation of TAs in the selected tissues and whole blood, 0.5 g homogenized tissues (T18 digital ULTRA-TURRAX, IKA, Germany) or 0.1 g blood samples (wet weight) were digested by adding the mixture of 6 ml 70% HNO_3_, 2 ml 30% HClO_4_ and 4 ml ammonium oxalate, respectively (MARS6 microwave digestion system, CEM Corporation, NC, United States). Thereafter, 1 ml thiourea-ascorbic acid mixture (50 g L^−1^) was added to the digested samples in centrifuge tubes and diluted with deionized water to reach 50 ml. The obtained mixture is then analyzed by HG-AFS.

To determine the bioaccumulation of diverse arsenic metabolites in the selected tissues, whole blood, or plasma samples, 100 μL homogenate solution of tissues (0.2 g tissues: 3 ml deionized water) or 0.1 g whole blood samples were mixed with 10 μL 5% ammonia water and 200 μL 30% H_2_O_2_ for digestion overnight at room temperature, respectively. After that, 50 μL 20% HClO_4_ was added to the 450 μL of pretreated tissue, blood samples, or thawed plasma samples, and the proteins were precipitated by centrifugation at 19,090 × *g* for 5 min at 4°C. Finally, 100 μL aliquots of the supernatant were injected into the HPLC-HG-AFS system.

### Statistical and Data Analysis

All data are presented as mean ± standard error of the mean (SEM). Statistical analyses and graphical presentations were performed with GraphPad Prism, Version 8.0.2 (GraphPad Prism software, San Diego, CA, United States). The significance of differences was assessed using either an unpaired t-test or one-way analysis of variance (ANOVA), and *p*-values less than 0.05 or 0.01 were considered statistically significant.

Haiguang HG-AFS Workstation (version 2.0.01) was used for data acquisition and analysis. Drug and Statistics (DAS) software (Version 3.0, Shanghai University of Traditional Chinese Medicine; Shanghai, China), a non-compartmental statistical model, was used to calculate the pharmacokinetic parameters. The relative tissue weights were calculated using the following equation:

$$\text{Tissue}\;\text{somatic}\;\text{indices}\%=\frac{\text{tissue}\;\text{weight}}{\text{body}\;\text{weight}}\times 100\text{.}$$

## Results

**FIGURE 1:**
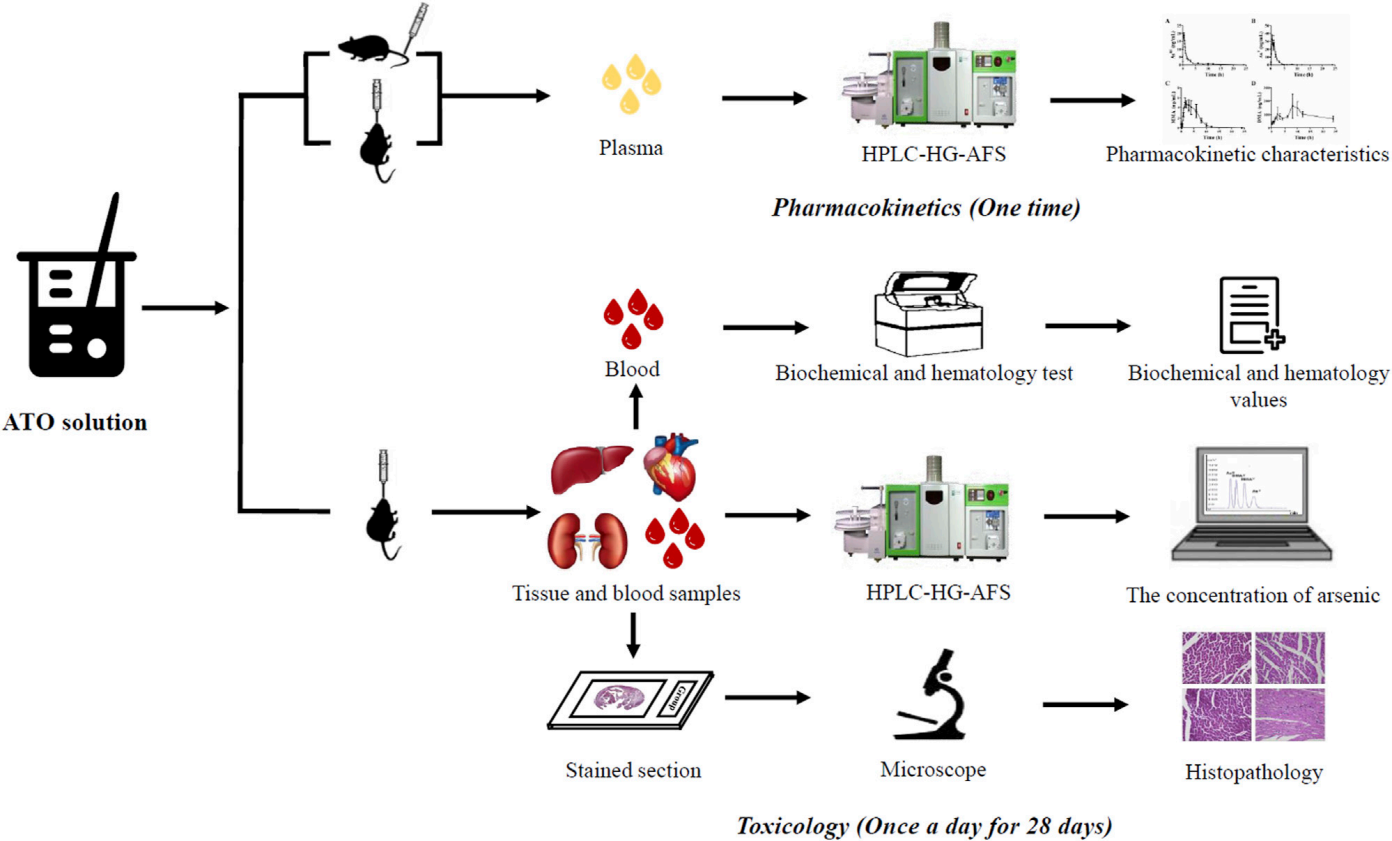
Pharmacokinetics and toxicology study in rats administered with oral ATO.

The flowchart of the pharmacokinetics and toxicology study of oral ATO are summarized in Figure 1.

### Pharmacokinetic Study by Single Intragastric and Intravenous Administration of Arsenic Trioxide

#### Pharmacokinetic Profiles of Arsenic Metabolites in Plasma

**FIGURE 2:**
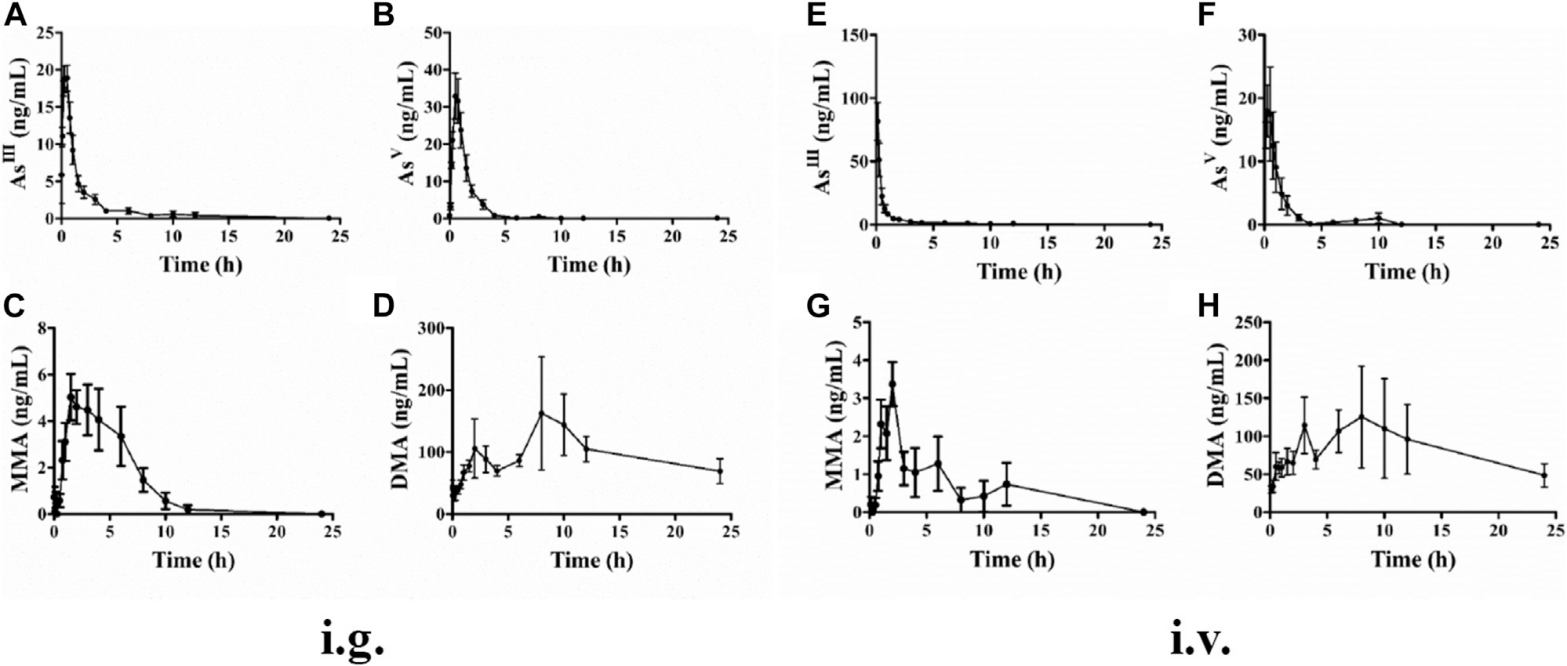
Mean plasma concentration-time curves of rats As^III^, As^V^, MMA^V^, and DMA^V^ after intragastric **(A–D)** and intravenous **(E–H)** administration to a single dose (1 mg kg^−1^). Data were shown as mean ± SEM. (*n* = 5 for each group).

A total of five replicates (corresponding to five rats) were measured for each time point, and the mean concentration was used to establish the pharmacokinetic time curves. The plasma concentration-time curves of the four arsenic metabolites after i.g. administration of ATO solution are shown in Figure 2. As^III^ was detected at 10 min after i.g. administration and 5 min after i.v. administration of ATO, which suggests a rapid absorption of As^III^ in the blood. As^V^ was detected within 8 h for both i.g. and i.v. administration groups. MMA^V^ appeared at 0.5 h after ATO administration and with concentration remaining stable at a relatively low level until at least 12 h. The predominant metabolite, DMA^V^, was found at high levels and could be detected from 0.5 to 24 h after ATO administration.

#### Pharmacokinetic Parameters of Arsenic Metabolites in Plasma

The mean pharmacokinetic parameters of plasma arsenic metabolites based on the non-compartmental model are summarized in Table 1 for i.g. and i.v. administration of ATO solution at a single dose of 1 mg kg^−1^. The maximum plasma concentrations (C_max_), area under the curve (AUC_0−t_) values and time to maximum concentration (T_max_) of As^III^ were 21.01 ± 0.89 vs. 81.49 ± 14.90 ng ml^−1^, 45.77 ± 16.91 vs. 56.49 ± 11.14 ng h^−1^ ml^−1^, and 0.22 ± 0.08 vs. 0.13 ± 0.01 h, respectively. The absolute oral bioavailability of As^III^ was 81.03%, which was calculated by dividing AUC_0-t_ of intragastrical administration by AUC_0-t_ of i.v. administration. Meanwhile, after the i.g. and i.v. administration, As^V^ was reached C_max_ at 33.93 ± 6.16 vs. 21.15 ± 6.54 ng ml^−1^, with AUC_0−t_ values of 56.14 ± 13.36 vs. 35.83 ± 9.62 ng h^−1^ml^−1^, respectively. As for MMA^V^, the AUC_0−t_ values and C_max_ of i.v. and i.g. administration were the lowest among the measured arsenic metabolites. Moreover, The C_max_ of DMA^V^ after i.v. administration was similar to the i.g. counterparts. The mean residence time (MRT) of DMA^V^ was longer than As^III^, As^V^, MMA^V^ with measurements of 11.41 ± 0.83, 5.21 ± 3.68, 1.51 ± 0.26, and 3.89 ± 0.39 h, respectively.

**TABLE 1:** Pharmacokinetic parameters of arsenic metabolites after a single intragastrical (i.g.) and intravenous (i.v.) administration of 1 mg kg^−1^ ATO in rats.

	As^III^		As^V^		MMA		DMA	
	i.g.	i.v.	i.g.	i.v.	i.g.	i.v.	i.g.	i.v.
C_max_ (ng ml^−1^)	21.01 ± 0.89	81.49 ± 14.90	33.93 ± 6.16	21.15 ± 6.54	6.40 ± 0.93	3.85 ± 0.43	264.89 ± 78.59	203.74 ± 52.87
T_max_ (h)	0.22 ± 0.08	0.13 ± 0.01	0.60 ± 0.06	0.28 ± 0.06	2.60 ± 0.86	2.50 ± 0.89	9.10 ± 4.08	4.60 ± 1.87
t_1/2α_ (h)	0.39 ± 0.10	0.14 ± 0.03	0.44 ± 0.05	0.49 ± 0.05	—	—	—	—
MRT (h)	5.21 ± 3.68	1.42 ± 0.45	1.51 ± 0.26	5.78 ± 3.66	3.89 ± 0.39	3.53 ± 0.73	11.41 ± 0.83	10.26 ± 0.43
CL (L h^−1^ kg^−1^)	35.14 ± 12.38	13.32 ± 3.08	23.83 ± 5.46	37.77 ± 8.17	—	—	—	—
AUC_0-t_ (ng h^−1^ ml^−1^)	45.77 ± 16.91	56.49 ± 11.14	56.14 ± 13.36	35.83 ± 9.62	28.96 ± 6.35	17.27 ± 6.55	2,303.78 ± 444.81	2013.48 ± 752.57
AUC_0-∞_ (ng h^−1^ ml^−1^)	48.52 ± 16.55	77.69 ± 22.97	57.07 ± 13.43	39.40 ± 8.83	36.15 ± 5.85	40.63 ± 12.71	3,391.61 ± 852.02	3,115.26 ± 1,222.93
F (%)	81.03							

Data were presented as mean ± SEM, *n* = 5. C_max_, maximum concentration in plasma; T_max_, time to reach the C_max_; t_1/2α_, terminal elimination half-life; MRT, mean residence time; CL, clearance rates.; AUC, area under the curve; F, absolute bioavailability.

—: indicates the concentrations of metabolites were below the limit of detection and cannot be further calculated.

### Toxicity Profiles and Tissue Bioaccumulation of Total Arsenic and Arsenic Metabolites Following Oral Arsenic Trioxide For 28 Days

#### Clinical Observation

Rats were monitored daily for any behavioral or clinical signs of toxicity. The rats in the control group and ATO groups at doses of 2 and 8 mg kg^−1^ d^−1^ appeared healthy for the duration of the study with no observed changes in mental state, behavior, or defecation. However, the rats in the 20 mg kg^−1^ d^−1^ ATO group exhibited slow action, rough and dull fur starting from the 14th day, which was aggravated continuously until the end of the study. None of the rats died during the experiment.

#### Body Weight and Tissue Somatic Indices

**FIGURE 3:**
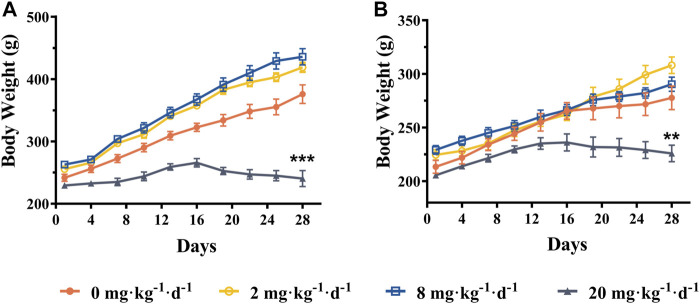
Mean body weight of male rats **(A)** and female rats **(B)** following intragastric administration of ATO for 28 days, respectively (mean ± SEM, *n* = 6 for each group). ** indicates statistically different from the control group (0 mg kg^−1^ d^−1^) at the 0.01 level.

As shown in Figure 3, the mean body weight was increased steadily in ATO treated groups (2 and 8 mg kg^−1^ d^−1^) compared with the control group, and no significant changes were noted (*p* > 0.05). However, the body weight of the rats in the 20 mg kg^−1^ d^−1^ group was significantly lower than that of the control group (*p* < 0.01). As presented in Table 2, the relative tissue somatic indices for the liver, kidney, and heart showed a slight decrease in the 2 and 8 mg kg^−1^ d^−1^ groups compared with the control groups (*p* > 0.05). Specifically, the relative heart, kidney, and liver somatic indices of male rats in the 20 mg kg^−1^ d^−1^ group were increased significantly compared to the control group (*p* < 0.05), but no significant changes were observed in the 20 mg kg^−1^ d^−1^ female group.

**TABLE 2:** Tissue somatic indices of rats following oral administration of ATO for 28 days.

Tissue	Male				Female			
	0 mg kg^−1^ d^−1^	2 mg kg^−1^ d^−1^	8 mg kg^−1^ d^−1^	20 mg kg^−1^ d^−1^	0 mg kg^−1^ d^−1^	2 mg kg^−1^ d^−1^	8 mg kg^−1^ d^−1^	20 mg kg^−1^ d^−1^
Liver	3.61 ± 0.20	3.73 ± 0.23	3.55 ± 0.23	4.73 ± 0.24^a^	4.03 ± 0.12	4.17 ± 0.29	3.91 ± 0.12	5.12 ± 0.23^b^
Kidney	0.41 ± 0.02	0.37 ± 0.02	0.37 ± 0.02	0.53 ± 0.03^b^	0.41 ± 0.01	0.39 ± 0.01	0.38 ± 0.01	0.44 ± 0.02
Heart	0.38 ± 0.01	0.38 ± 0.01	0.36 ± 0.01	0.48 ± 0.02^b^	0.43 ± 0.01	0.42 ± 0.02	0.39 ± 0.01	0.44 ± 0.02

Data were expressed as mean ± SEM, *n* = 6.

a Indicates a statistical difference compared to the 0 mg kg^−1^ d^−1^ group at the 0.05 level.

b Indicates a statistical difference compared to the 0 mg kg^−1^ d^−1^ group at the 0.01 level.

#### Biochemistry and Hematology Examination

The measured values for biochemistry and hematology examination performed after 28 days of treatment with oral ATO at doses of 2, 8, and 20 mg kg^−1^ d^−1^ are presented in Table 3. Total protein (TP) remained at the control level for both male and female rats at the three doses tested. A significant increase of ALT, AST, and HGB in comparison to the control group was observed for male rats at the three ATO doses tested but for the female rats, it was only significantly increased at the 8 mg kg^−1^ d^−1^ ATO dose for ALT and AST and 20 mg kg^−1^ d^−1^ ATO dose for HGB (*p* < 0.05). Both male and female rats presented a significant increase in GLU concentration compared to the control group for the 20 mg kg^−1^ d^−1^ ATO dose (*p* < 0.01). A significant elevation in WBC levels was observed for the female group at the three ATO tested but only at 20 mg kg^−1^ d^−1^ ATO for the male group (*p* < 0.05). RBC count remained stable in the female rats for the three ATO doses but was significantly higher than the control level in the male rats at the three ATO doses tested (*p* < 0.05). NeU count levels decreased only in male rats at the 2 mg kg^−1^ d^−1^ ATO dose but increased in both male and female rats at the 20 mg kg^−1^ d^−1^ ATO dose. The PLT levels followed the same pattern in both male and female rats with a significant decrease observed for the 2 mg kg^−1^ d^−1^ ATO dose and a significant increase observed for the 20 mg kg^−1^ d^−1^ ATO dose (*p* < 0.01).

**TABLE 3:** Hematology and serum biochemical values of rats following oral administration of ATO for 28 days.

Items	Male				Female			
	0 mg kg^−1^ d^−1^	2 mg kg^−1^ d^−1^	8 mg kg^−1^ d^−1^	20 mg kg^−1^ d^−1^	0 mg kg^−1^ d^−1^	2 mg kg^−1^ d^−1^	8 mg kg^−1^ d^−1^	20 mg kg^−1^ d^−1^
TP (g L^−1^)	53.63 ± 1.75	51.15 ± 1.50	58.68 ± 2.90	56.05 ± 1.18	52.28 ± 2.33	54.37 ± 1.54	59.83 ± 2.73	53.8 ± 0.91
ALT (U L^−1^)	43.5 ± 1.43	59.33 ± 2.90^a^	72.17 ± 3.91^a^	50.17 ± 0.87^a^	56.83 ± 2.46	61 ± 1.95	69.17 ± 1.66^a^	52.67 ± 10.04
AST (U L^−1^)	115.17 ± 4.04	136 ± 7.28^b^	164 ± 5.87^a^	139.83 ± 9.57^b^	116.33 ± 3.30	131.5 ± 7.27	166 ± 7.45^a^	148 ± 30.00
GLU (mmol L^−1^)	8.22 ± 0.95	ND	ND	14.38 ± 1.10^a^	5.70 ± 0.27	ND	ND	9.37 ± 0.64^a^
WBC (10^9^ L^−1^)	14.26 ± 1.65	13.88 ± 1.27	18.8 ± 1.45	26.55 ± 1.06^a^	12.26 ± 0.49	16.8 ± 1.23^a^	27.68 ± 3.94^a^	16.08 ± 1.16^b^
RBC (10^12^ L^−1^)	6.02 ± 0.17	6.74 ± 0.145^b^	7.70 ± 0.50^b^	8.01 ± 0.079^a^	6.01 ± 0.36	6.48 ± 0.37	6.32 ± 0.36	6.84 ± 0.27
NeU (10^9^ L^−1^)	3.03 ± 0.42	2.00 ± 0.10^b^	4.28 ± 0.47	9.69 ± 1.70^a^	2.86 ± 0.21	2.41 ± 0.25	3.83 ± 0.57	6.15 ± 1.31^b^
HGB (g L^−1^)	115.5 ± 4.40	136.67 ± 4.28^a^	145.67 ± 6.47^a^	151.17 ± 1.42^a^	110.5 ± 6.91	128.5 ± 5.16	120.33 ± 6.40	131 ± 5.35^b^
PLT (10^9^ L^−1^)	584.67 ± 11.46	583.5 ± 16.49	517.33 ± 13.10^a^	815.83 ± 55.18^a^	550.17 ± 19.24	537.67 ± 12.55	459.67 ± 18.75^a^	823.5 ± 66.81^a^

Data were expressed as mean ± SEM, *n* = 6. TP, total protein; ALT, alanine aminotransferase; AST, aspartate aminotransferase; GLU, glucose; WBC, white blood cells; RBC, red blood cells; NeU, neutrophils absolute value; HGB, hemoglobin; PLT, platelet; ND, indicates the data were not detected.

a Indicates a statistical difference compared to the 0 mg kg^−1^ d^−1^ group at the 0.01 level.

b Indicates a statistical difference compared to the 0 mg kg^−1^ d^−1^ group at the 0.05 level.

#### Histopathological Evaluation

**FIGURE 4:**
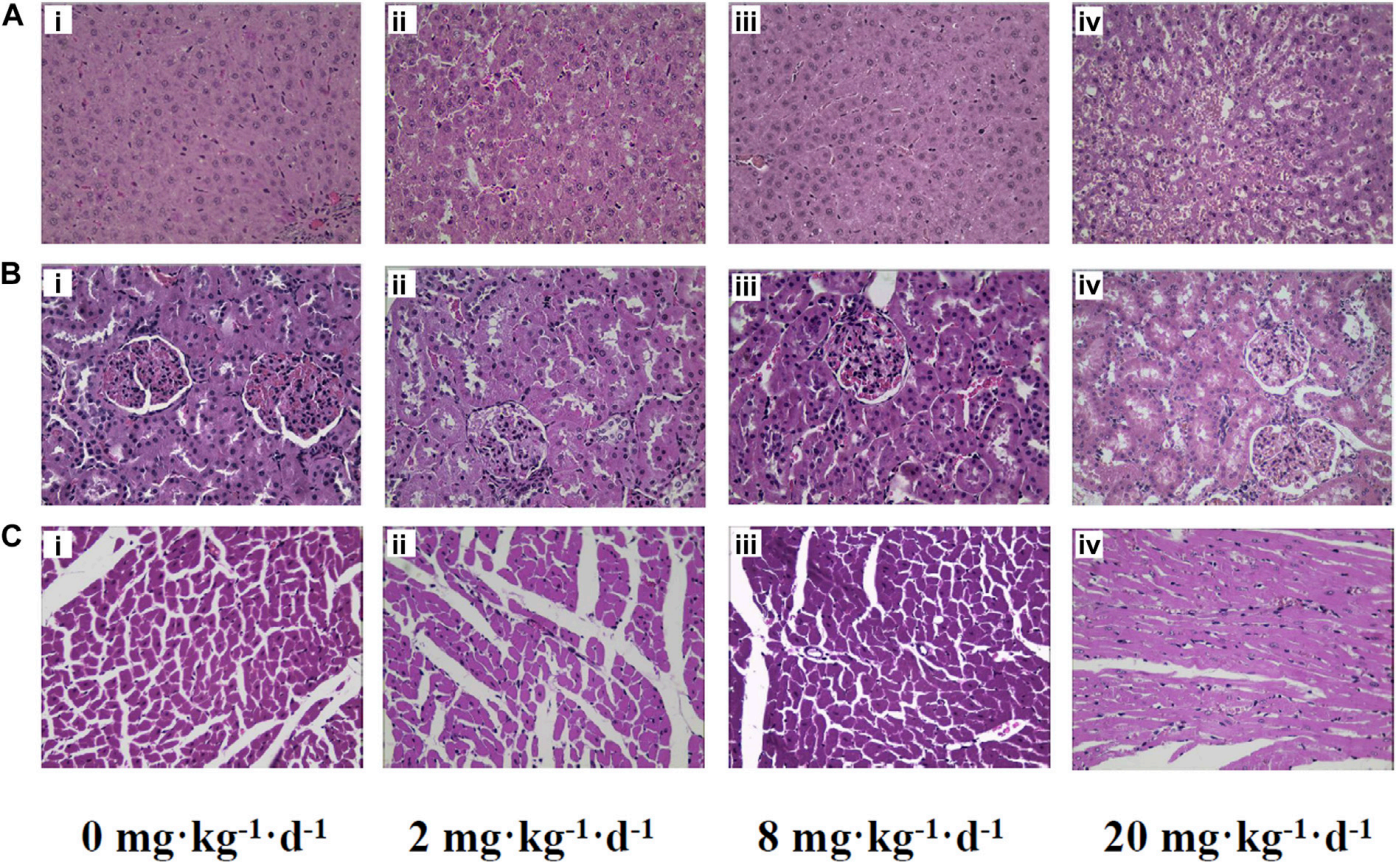
Representative histopathology slices of rats liver **(A)**, kidney **(B)**, heart **(C)** following the intragastric administration of ATO for 28 days **(i)** 0 mg kg^−1^ d^−1^, control group; **(ii)** 2 mg kg^−1^ d^−1^ group; **(iii)** 8 mg kg^−1^ d^−1^ group; **(iv)** 20 mg kg^−1^ d^−1^ group (H&E Stain, magnification ×400).

Histopathological examination was performed to determine the extent of organ toxicity as a consequence of the ATO treatment. Representative histopathology images of the organs (liver, kidney, and heart) are illustrated in Figure 4. No obvious changes have been found in the morphological characteristics of the organs in the control group, which showed obvious cell nuclei, sinusoid, and delimited cytoplasm (Figures 4Ai–Ci). In contrast, the rats treated with 2 and 8 mg kg^−1^ d^−1^ ATO presented slight histopathological changes of organs characterized by occasional vacuolization and focal necrosis of the liver as well as hydropic degeneration of some renal tubules, (Figures 4Aii,iii,Bii,iii). Meanwhile, the histopathological changes of liver and kidney tissue samples in 20 mg kg^−1^ d^−1^ showed a large number of apoptotic hepatocytes, congestion of the liver tissue, and severe deformation of renal tubular epithelial cells (Figures 4Aiv,Biv). Furthermore, occasional mild inflammatory infiltration was observed in the heart tissue of the 2, 8, and 20 mg kg^−1^ d^−1^ ATO group, but there was no significant difference in the degree of infiltration among the three groups (Figures 4Cii–iv).

#### Total Arsenic and Arsenic Metabolites Determination in Organ Samples

**FIGURE 5:**
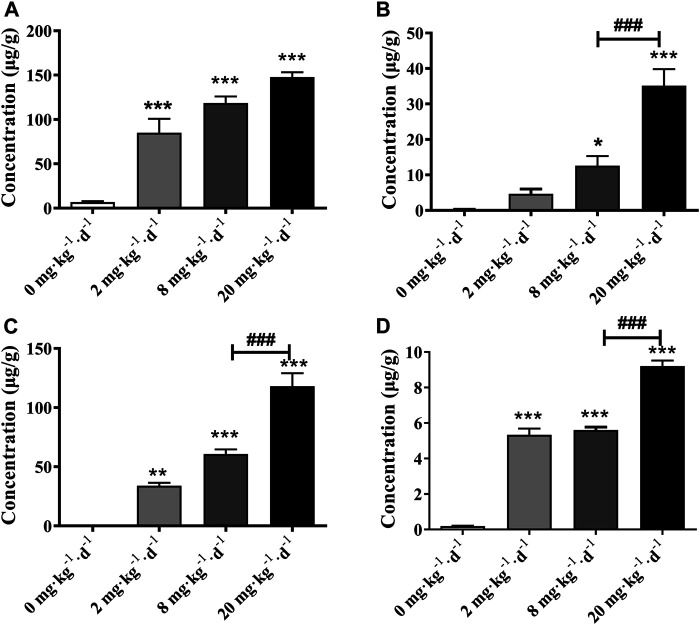
Total arsenic concentration in rat tissues following 28 days intragastric administration of different doses of ATO. Total arsenic concentration in the blood **(A)**, liver **(B)**, kidney **(C)**, heart **(D)**. Asterisks indicate statistical differences compared to the control group (0 mg kg^−1^ d^−1^) at *p* < 0.05 (*); *p* < 0.01 (**); *p* < 0.001 (***). ^###^: statistical difference compared to the 8 mg kg^−1^ d^−1^ group (*p* < 0.001).

**FIGURE 6:**
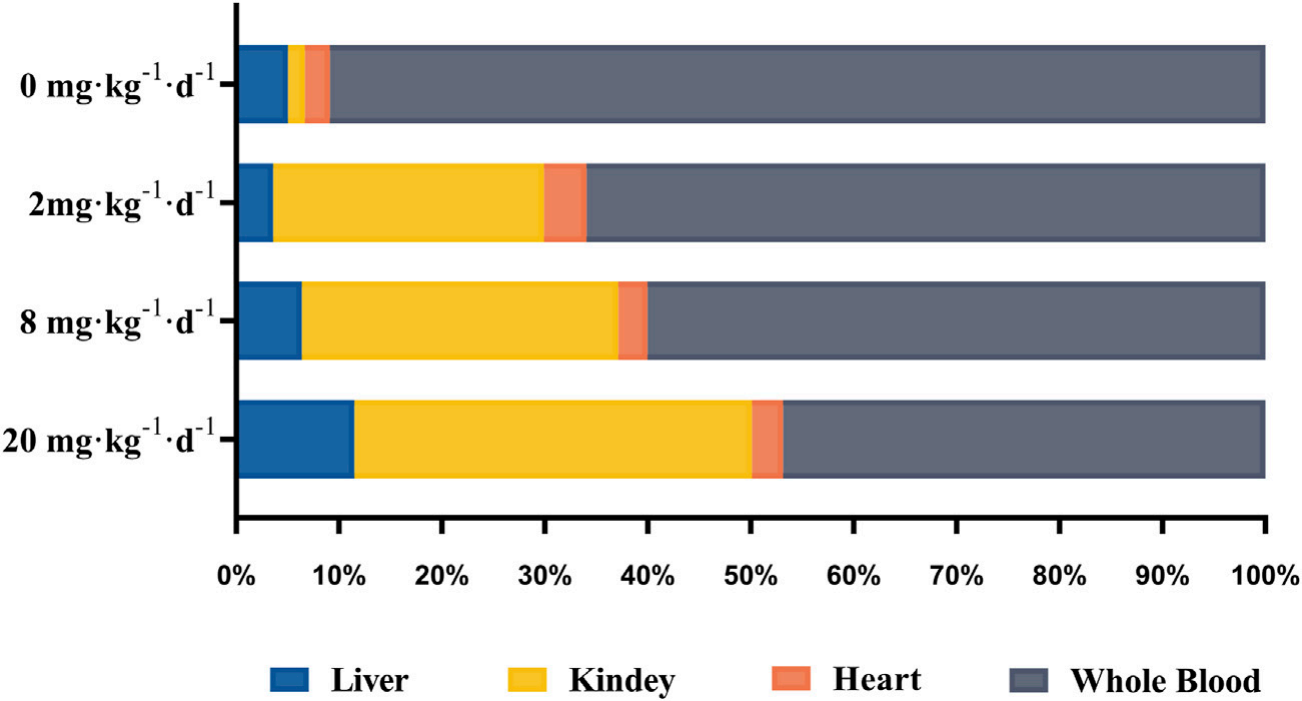
The distribution of total arsenic in whole blood and tissue samples after intragastric administration of ATO for 28 days.

The tissue bioaccumulation profiles of TAs in rats after intragastric administration of different doses of ATO for 28 days are charted in Figure 5. The concentration of TAs in whole blood, liver, kidney, and heart tissue of rats treated with 2, 8, and 20 mg kg^−1^ d^−1^ was significantly higher than that of the control group (*p* < 0.01). The bioaccumulation level of TAs exhibited an obvious dose-dependent increase which is positively correlated with the dose of ATO administrated. Additionally, as shown in Figure 6, the bioaccumulation of TAs in the different treated groups of tissues was arranged consistently as follows: whole blood > kidney > liver > heart. Results showed that with the increase of ATO dose from 2 to 20 mg kg^−1^ d^−1^, the proportion of TAs in whole blood decreased from 65.95 to 46.87%, while that in liver and kidney increased from 3.59 to 11.49%, and 26.32–38.63%, respectively. However, the content of TAs in heart tissue remained at a stable and relatively low level with a proportion maintained at 2–4%.

Moreover, four metabolites of As^III^, As^V^, MMA, DMA were detected in the liver, kidney, and heart tissues, while only DMA was detected in whole blood (Table 4). The concentration of arsenic metabolites in liver tissue was DMA > iAs > MMA. The proportion of DMA relative to the arsenic metabolites decreased from 95.08% for the 2 mg kg^−1^ d^−1^ dose and 95.04% for the 8 mg kg^−1^ d^−1^ dose to 68.55% for the 20 mg kg^−1^ d^−1^ dose, while the proportion of iAs increased from 3.74% for the 2 mg kg^−1^ d^−1^ dose and 3.88% for the 8 mg kg^−1^ d^−1^ dose to 27.72% for the 20 mg kg^−1^ d^−1^ dose. In particular, DMA was the predominant metabolite in the kidney for the 2 mg kg^−1^ d^−1^ ATO group with a proportion of 74.93% among arsenic metabolites followed by MMA at a proportion of 23.63%. However, at the dose of 8 and 20 mg kg^−1^ d^−1^, MMA was the predominant metabolite in the kidney, and the proportion of MMA increased to 60.36 and 72.70%, while DMA was decreased to 37.21 and 24.68%, respectively. Furthermore, the arsenic metabolite proportion in the rat heart tissue was similar for the three doses tested with ATO, the DMA was the predominant metabolite and at a proportion of more than 97%, while MMA and iAs remained at low levels.

**TABLE 4:** Tissue and blood arsenic metabolites concentration following oral administration of ATO for 28 days.

Samples	Dose (mg kg^−1^ d^−1^)	iAs		MMA		DMA	
		Male	Female	Male	Female	Male	Female
Liver	0	70.61 ± 22.90	34.39 ± 7.56	—	—	217.35 ± 119.83	337.67 ± 27.18
	2	645.71 ± 64.30	324.78 ± 139.45	172.72 ± 1.30	83.11 ± 4.15	10,422.87 ± 403.15	14,256.67 ± 261.09^b^
	8	489.18 ± 55.81	487.35 ± 20.21	136.40 ± 6.01	—	15,508.59 ± 530.18	8,380.42 ± 128.61^a^
	20	4,211.67 ± 33.33	15,460.67 ± 177.78^a^	1,400.67 ± 21.87	1,278.67 ± 47.26	26,270.00 ± 602.50	23,574.00 ± 1,284.21
Kidney	0	58.17 ± 18.56	—	57.95 ± 31.48	—	793.34 ± 54.42	225.34 ± 10.83^a^
	2	124.07 ± 8.09	270.27 ± 69.73	5,283.73 ± 156.08	1,108.85 ± 137.82^a^	10,609.93 ± 360.73	9,662.85 ± 568.05
	8	984.42 ± 244.90	477.48 ± 229.07	18,151.13 ± 484.70	18,179.78 ± 121.44	10,731.26 ± 277.41	11,666.54 ± 200.98
	20	687.33 ± 297.71	3,417.00 ± 330.58^b^	32,502.33 ± 1873.04	38,486.00 ± 1874.27	16,867.33 ± 143.06	21,718.67 ± 546.80^b^
Heart	0	—	—	—	—	155.51 ± 15.82	211.62 ± 46.44
	2	60.80 ± 20.66	56.16 ± 18.72	53.20 ± 19.25	59.68 ± 21.89	5,352.48 ± 172.13	5,822.19 ± 215.41
	8	32.14 ± 3.71	39.04 ± 12.79	24.54 ± 8.22	29.53 ± 19.29	5,438.74 ± 709.09	5,139.04 ± 308.69
	20	153.88 ± 23.29	165.91 ± 24.62	52.66 ± 7.03	74.27 ± 3.35	9,099.41 ± 265.57	8,867.07 ± 655.51
Whole Blood	0	—	—	—	—	5,374.33 ± 2,256.84	7,699.00 ± 776.59
	2	—	—	—	—	81,583.33 ± 22,153.67	88,383.33 ± 27,105.19
	8	—	—	—	—	112,652.00 ± 3,955.00	110,768.00 ± 9,395.68
	20	—	—	—	—	144,592.47 ± 11,027.18	142,092.57 ± 7,269.17

Data were expressed as mean ± SEM, *n* = 6.

—: Indicates the concentration of arsenic metabolites was below the limit of detection.

a Indicates a statistical difference compared to the 0 mg kg^−1^ d^−1^ group at the 0.01 level.

b Indicates a statistical difference compared to the 0 mg kg^−1^ d^−1^ group at the 0.01 level.

## Discussion

Oral ATO is highly efficient for APL treatment (Gill et al., 2018; Kumana et al., 2020; Ravandi et al., 2020). Unlike the conventional all-trans retinoic acid and chemotherapeutic drugs, ATO not only can induce CR consistently though as a single agent but also showed promising anticancer activities on other malignancies (Emadi and Gore, 2010; Mathews et al., 2010; Khairul et al., 2017). However, long-term and high-dose medication of ATO will inevitably cause bioaccumulation of arsenic metabolites, which are associated with adverse effects (Wang et al., 2013; Xiang et al., 2019). Previous studies have suggested that appropriate dosage can reduce the incidence of potential adverse effects (Lo-Coco et al., 2013; Gill et al., 2018; Kumana et al., 2020), and proposed several possible mechanisms of organ toxicity, including the promotion of oxidative damage, disturbance of ion channel balance, alteration of DNA methylation, and so on (Alamolhodaei et al., 2015; Hu et al., 2018; Chang and Singh, 2019). However, the dosage of the organ toxicity induced by oral ATO as well as the toxicologic and therapeutic potential of different arsenic metabolites in the treatment of APL and other cancers have not yet been fully clarified. In this context, the results of the present study is the first time to illuminate the pharmacokinetic characteristics, tissue bioaccumulation, and subchronic toxicity profile of arsenic metabolites following successive oral ATO administration.

Pharmacokinetics is an important drug safety evaluation method that has been applied in human health risk assessment (Walker, 2004). A previous study only focused on the pharmacokinetic profiles and bioavailability of TAs in APL patients treated with oral ATO but its main pharmacologically active metabolite of ATO is As^III^ (Firkin, 2012). It is known that As^III^ undergoes a series of redox methylation reactions *in vivo* to form As^V^, and diverse methylated metabolites (Cullen, 2014). Meanwhile, given the fact that the different arsenic metabolites have unique therapeutic or toxicologic potentials (Khaleghian et al., 2014; Khairul et al., 2017; Hirano, 2020). Thus, there has been growing recognition that elucidates the pharmacokinetics of diverse plasma arsenic metabolites level was more critical for the treatment and risk assessment of ATO treatment (Shi et al., 2019; Ravandi et al., 2020). Furthermore, oral and i.v. administration of ATO are two different drug delivery patterns that may generate different proportions and concentrations of arsenic metabolites *in vivo*, resulting in toxicity differences.

In the present study, the pharmacokinetic parameters and profiles of As^III^, As^V^, MMA^V^, and DMA^V^ in plasma of male SD rats after i.g. and i.v. administration of 1 mg kg^−1^ ATO are presented in Figure 2 and Table 1, respectively. As shown in Figure 2, the time of the first detection and T_max_ of As^III^ in plasma after i.g. and i.v. administration was 10 vs. 5 min, 0.22 ± 0.08 h vs. 0.13 ± 0.01 h, respectively. The absolute oral bioavailability of As^III^ was approximately equal to 81.03%, which demonstrated the fact that orally administered ATO was well absorbed (Ravandi et al., 2020), and the results were higher than the results of the previous study (81.03 vs. 60.9%) (Shi et al., 2019). It is noteworthy that the C_max_ of i.g. and i.v. administration of As^III^ was 21.01 ± 0.89 vs. 81.49 ± 14.90 ng ml^−1^, respectively. Considering that a previous study proposed that instantaneous high concentration of ingested arsenic can cause QTc interval prolongation (Siu et al., 2006) to some extent, it seems that oral ATO administration could potentially reduce the incidence of adverse cardiac events. Thus, oral ATO appears to be safer than i.v. administration ATO in addition to its similar therapeutic effect. Furthermore, it was also observed that As^III^ was rapidly oxidized to As^V^, which was first detected at 10 min and reached a C_max_ of 33.93 ± 6.16 ng ml^−1^ at 0.60 ± 0.06 h. In addition, MMA^V^ as an intermediate metabolite appeared at 0.5–8 h and remained at a stable and relatively low level. As for the predominant metabolite, the elimination of DMA^V^ from plasma was lower than the As^III^, with observable levels present at 24 h (Figure 2 and Table 1). As shown in Figure 2, the plasma DMA^V^ unexpectedly showed a bimodal phenomenon, we speculated that it may be attributed to the enterohepatic cycle of DMA or a certain difference in arsenic methylation metabolism among experimental rats (Kala et al., 2000).

Recently, mounting evidence supports the view that methylation metabolism plays a pivotal role in arsenic toxicity (Wang et al., 2013; Khairul et al., 2017; Kuo et al., 2017; Hirano, 2020). Thus, understanding the tissue bioaccumulation and toxicity profile of arsenic metabolites in animals is a preliminary step towards illuminating the possible toxicities in human organs for sub-chronic oral ATO treatment. In the present study, HPLC-HG-AFS was applied to determine the levels of TAs, iAs and the methylated metabolites in the blood, liver, kidney, and heart of rats, at the same time, histopathology, biochemistry, and hematology examinations were also carried out to elaborate on the potentially biological or toxicological outcomes of orally administrated ATO at 0, 2, 8, and 20 mg kg^−1^ d^−1^ for 28 days.

As indicated in previous epidemiological studies, the relationship between arsenic exposure and metabolic abnormalities remains obscure (Farkhondeh et al., 2019). In the present study, the rat mean body weight increased steadily in 2 and 8 mg kg^−1^ d^−1^ ATO groups compared with the control group (*p* > 0.05). Specifically, the body weight of the 20 mg kg^−1^ d^−1^ group was significantly lower than that of the control group, as observed after the second week of administration (*p* < 0.01) (Figure 3). Meanwhile, the rats in the 20 mg kg^−1^ d^−1^ ATO group were showed slow action, rough, dull fur, and a significant increase of GLU in both male and female group compared to the 0, 2, and 8 mg·kg^−1^·d^−1^ ATO group (*p* < 0.01) (Table 3). Our findings are consistent with the previous viewpoint that arsenic exposure could exert negative effects on body weight at a high dose level and increase the risk of diabetes (Farkhondeh et al., 2019; Lucio et al., 2020). Furthermore, the levels of ALT, AST, WBC, NeU, and PLT showed an obvious upward trend that correlated with the increase of the ATO dose (Table 3) (*p* < 0.01). This suggests that oral ATO may lead to liver injury at a high dose level, which is consistent with observations from previous clinical studies (Kumana et al., 2020; Ravandi et al., 2020; Gill et al., 2018; Sasijareonrat et al., 2020). There was a significant difference between the male and female rats in terms of changes in biochemistry and hematology values, which may be attributed to sex differences in the arsenic methylation capacity (Tables 3, 4) (Torres-Sánchez et al., 2016). Therefore, even though oral ATO has been shown to be highly effective and well-tolerated in low-risk APL patients, our results suggest that periodical monitoring of hematological and biochemical parameters is still recommended.

On the other hand, it is generally believed that prolonged and excessive exposure to ATO may cause the bioaccumulation of arsenic compounds in vital organs with ensuing toxic effects (Wang et al., 2013; Xiang et al., 2019). After the oral ATO for 28 days, the liver, kidney, heart somatic indices of male rats in the 20 mg kg^−1^ d^−1^ group increased significantly compared to the 0, 2, and 8 mg kg^−1^ d^−1^ groups (*p* < 0.05), while there were no significant changes in the heart and kidney somatic indices of female rats (Table 2). We assumed that the increase of the tissue index may be associate with the inflammatory response caused by the bioaccumulation of specific arsenic metabolites or tissue edema induced by high-dose arsenic exposure (Figure 4) (Li et al., 2017). The TAs levels in the whole blood and all tested organs had significant increases with the elevation of doses of ATO and showed a significant dose-dependent manner (*p* < 0.01) (Figure 5).

As shown in Figure 6, the bioaccumulation of TAs in different treated groups of tissues was arranged consistently as follows: whole blood > kidney > liver > heart. This result differs from that of a previous study, which could be attributed to the differences in the experimental animal species, ATO dose, drug pretreatment methods, or sample analysis methods (Kenyon et al., 2008). As shown in Table 4, DMA is the only detected arsenic metabolite in whole blood, and while there are four metabolites measurable in liver, kidney, and heart tissues (As^III^, As^V^, MMA, DMA). Specifically, the DMA was the predominant metabolite in the kidney at the dose of 2 mg kg^−1^ d^−1^ ATO group, while MMA was the predominant metabolite in the kidney at the dose of 8 and 20 mg kg^−1^ d^−1^ ATO group, which showed a similar distribution tendency compared to previous studies (Kenyon et al., 2008; Yi et al., 2020). Possible explanations are the induction of specific binding transporter proteins and failure of a normal efflux mechanism, such as the lack of multidrug resistance gene protein, P-glycoprotein, and aquaglyceroporin-9 (Kenyon et al., 2008; Carbrey et al., 2009). The concentration of arsenic metabolites in the heart and liver tissue was DMA > iAs > MMA (Table 4). Although arsenic metabolites also bioaccumulated in the hearts of rats treated with 2, 8, and 20 mg kg^−1^ d^−1^ ATO, no significant cardiac pathological damage was observed (Figure 4C). These results further confirm that oral ATO is well tolerated and has low cardiac toxicity (Siu et al., 2006; Gill et al., 2018; Ravandi et al., 2020).

Increasing evidence indicates that the liver is not only the primary site for arsenic methylation but also a potential target of arsenic toxicity (Lu et al., 2019; Wang et al., 2013; Hu et al., 2018). Except for elevations of ALT and AST, two markers of liver injury, the histopathologic examination in the present study showed the occurrence of congestion and focal necrosis in liver tissue at the moderate-high dose level of ATO exposure (Figure 4A). As shown in Table 4, DMA was the predominant metabolite in liver tissue regardless of the oral dose of ATO administered (account for more than 68%), which was consistent with results from our clinical studies, and further confirms previous studies suggesting that a high level of DMA is an important predisposing factor for liver injury in ATO treatment (Wang et al., 2013; Lu et al., 2019). Moreover, the proportion of DMA in metabolites decreased from 95.08 to 68.55% with the increase of the dose, while the proportion of iAs increased from 3.74 to 27.72%. This could potentially be a consequence of the inhibition of methyltransferase by excessive arsenic impairing the capacity of arsenic methylation metabolism (Marafante and Vahter, 1984).

Although the 28 days repeated oral toxicity examination in the present study provides preliminary evidence for the assessment of the toxicity caused by repeated administration in a short period and the determination of the maximum dosage, there are certain differences between rodent animals and humans, and the extrapolation of experimental results to humans still has limitations. On the other hand, some differences in the metabolism of arsenic exist between rats and mice, and the pattern of tissue distribution in the mouse is more similar to that in humans (Vahter et al., 1984). Thus, the tissue distribution of arsenic metabolites in mice should be further investigated to elucidate the health risk of arsenic bioaccumulation during the APL patients treated with ATO. The analytical methods applied in this study are also not sufficient in distinguishing the oxidation state of the arsenic methylated metabolites (MMA^III^, MMA^V^, DMA^III^, and DMA^V^), due to the highly reactive and rapid conversion of MMA^III^ and DMA^III^. The existing analytic method is difficult to determine the trivalent methylated metabolites effectively in the blood and tissues. Whenever possible, distinguishing the bioaccumulation of specific trivalent and pentavalent arsenic metabolites in blood and tissues is highly desirable for elucidating the toxicologic and therapeutic mechanisms of ATO in the treatment of APL and other malignant tumors.

## Conclusion

Taken together, the results reported in the present study demonstrated that the bioaccumulation of arsenic and its methylated metabolites was organ-specific and dose-specific. On the other hand, it also suggests that the oral administration of ATO was well absorbed and tolerated. Although there are species differences between rats and humans, the results reported here provide a preliminary basis for sub-chronic safety and risk assessment of the oral ATO in APL treatment.

## Data Availability

The original contributions presented in the study are included in the article/Supplementary Material, further inquiries can be directed to the corresponding author.
